# Bacterial clearance reverses a skewed T-cell repertoire induced by *Salmonella* infection

**DOI:** 10.1002/iid3.60

**Published:** 2015-05-06

**Authors:** Jessica P Leyva-Rangel, Maria de los Angeles Hernández-Cueto, Carlos-Samuel Galan-Enriquez, Marcela López-Medina, Vianney Ortiz-Navarrete

**Affiliations:** 1 Doctorado en Ciencias Biomédicas Facultad de Medicina, UNAMMexico City, CP 045510, Mexico; 2 Departamento de Biomedicina Molecular, Centro de Investigación y Estudios Avanzados del Instituto Politecnico Nacional (CINVESTAV)Mexico City; 3 Hospital General la Raza, Instituto Mexicano del Seguro Social (IMSS)Mexico City

**Keywords:** Negative selection, positive selection, *Salmonella*, T-cell repertoire, thymus

## Abstract

*Salmonella typhimurium* invades the spleen, liver, and peripheral lymph nodes and has recently been detected in the bone marrow and thymus, resulting in a reduced thymic size and a decline in the total number of thymic cells. A specific deletion of the double-positive cell subset has been characterized, yet the export of mature T cells to the periphery remains normal. We analyzed *Salmonella* pathogenesis regarding thymic structure and the T-cell maturation process. We demonstrate that, despite alterations in the thymic structure, T-cell development is maintained during *Salmonella* infection, allowing the selection of single-positive T-cell clones expressing particular T-cell receptor beta chains (TCR-Vβ). Moreover, the treatment of infected mice with an antibiotic restored the normal thymic architecture and thymocyte subset distribution. Additionally, the frequency of TCR-Vβ usage after treatment was comparable to that in non-infected mice. However, bacteria were still recovered from the thymus after 1 month of treatment. Our data reveal that a skewed T-cell developmental process is present in the *Salmonella*-infected thymus that alters the TCR-Vβ usage frequency. Likewise, the post-treatment persistence of *Salmonella* reveals a novel function of the thymus as a potential reservoir for this infectious agent.

## Introduction

The thymus, a central lymphoid organ in which bone marrow emigrant thymocyte progenitor cells (ETPs) develop into mature CD4 and CD8 single-positive (SP) T cells, is also a target organ for infectious diseases 1–7. Under homeostatic conditions, newly arrived progenitors, termed double-negative (DN, CD4^−^CD8^−^) thymocytes, localize to the cortex region of the thymus to initiate sequential stages of maturation toward a specific T-cell phenotype. The selection of the beta chain, which forms the pre-T-cell receptor (pre-TCR) by combining with the pTα, is the first checkpoint that inhibits the generation of inappropriately rearranged TCRs 8–10. These receptors are subsequently submitted to positive and negative selection processes in the recognition of endogenous specific antigens presented by antigen-presenting cells (APCs) 11–13. As a result, thymocytes with TCRs that have weak or null engagement of endogenous peptides presented by major histocompatibility (MHC) molecules on the thymic APCs undergo programmed cell death, which is termed death by neglect. Thymocytes are positively selected in the cortex via an intermediate affinity recognition step and then subsequently localized to the medulla, where excessively strong TCR-peptide-MHC (pMHC) ligand interactions lead to negative selection. This deletion of thymocytes removes highly autoreactive cells from the T-cell repertoire, which egress to the periphery 14. Regulatory T cells (nTregs), which play a crucial role in the control of autoimmunity and tolerance to self-antigens, also originate in the thymus. It is thought that nTregs initially undergo the same selective pressure and developmental checkpoints as conventional T cells. However, nTregs are selected based on high-affinity recognition that is stronger than that required for conventional T-cell commitment but less than the threshold required for negative selection 15. As expected, intact, well-defined cortical, and medullary regions of the thymus represent an important niche for the maintenance of a proper T-cell developmental process 16–18. Morphological changes in this tissue are frequently attributed to thymic atrophy, which ultimately leads to organ involution 19,20. Nonetheless, several reports have identified residual thymic activity in various inflammatory models 3,21–24.

A general feature of an infected thymus is the severe apoptosis-related depletion of immature CD4^+^CD8^+^ double-positive (DP) thymocytes 4. However, during EBV infection, an increase in the proliferative response of infected thymocytes through interactions with CD21 has been observed 25. After infection with *Mycobacterium avium*, the bacteria disseminate to the thymus and promote the generation of T cells that retain their ability to reconstitute the periphery but are unable to sustain immunoprotection against a challenge from that same pathogen 26.

During *Salmonella* infection, thymic atrophy has been evaluated using attenuated and virulent strains. The infection of mice with live attenuated bacteria induces a temporary reduction in organ size and diminishes the percentages of all the thymocyte subsets while maintaining the thymic structure throughout the infection. In addition, the recovery of the thymus follows a time course that includes an increase in newly arrived ETPs and bacterial elimination 27. In contrast, mice infected with a virulent strain demonstrate a specific depletion of DP thymocytes that is associated with membrane damage and increased caspase-3 activity 28. However, this effect is not observed with the DN or SP populations, and the egress of mature T cells toward peripheral lymph node organs does not significantly change. Together, these data suggest that *Salmonella* infection induces damage to the structure of the thymus but maintains T-cell maturation.

In the present study, we evaluated the impact of *Salmonella typhimurium* infection on the structure of the thymus and the T-cell maturation process. We observed dose-dependent damage of the cortical and medullary regions of the thymi of mice infected with a virulent strain. Nevertheless, we recovered equal numbers of bacteria in the thymus irrespective of the initial dose administered, suggesting that the tissue damage depended more on the initial immune response than on the presence of the bacteria. Furthermore, we observed an increase in apoptosis but not a complete loss of all the thymic subsets, as opposed to a severe reduction in proliferation. In addition, we demonstrated the ongoing selection of DP thymocytes toward CD4 and CD8 SP T cells derived from particular T-cell receptor beta chain (TCR-Vβ) families. These alterations were reversed when the infected mice were treated with antibiotic; treating the infected mice for 1 month recovered normal cortical and medullary structures as well as normal ratios of all the thymocyte populations compared with the untreated controls. Likewise, the preferred selection of certain TCR-Vβ families was reversed, resulting in a normal TCR-Vβ usage frequency. Unexpectedly, we still recovered bacteria from the thymi of the treated mice following 1 month of antibiotic treatment. In summary, we demonstrated that *Salmonella* infection leads to an abnormal thymic structure and aberrant T-cell maturation processes that favor the selection of specific T-cell clones. Furthermore, controlling the systemic bacterial load with an antibiotic reestablishes a normal thymic structure and function despite a persistent low bacterial load in this organ.

## Materials and Methods

### Mice, bacteria, and immunization protocol

C57BL/6 mice were obtained from the CINVESTAV animal facility (Mexico City, Mexico). At 6–8 weeks of age, the mice were infected intraperitoneally (i.p.) with a specific dose of 50 or 500 bacteria consisting of wild-type live *Salmonella enterica* serovar Typhimurium 14028 or live attenuated *S. typhimurium* SL3261 AroA^−^ (obtained from Cesar Gonzalez Bonilla, Medical Research Unit in Immunology and Infectious Disease, Centro Médico Nacional La Raza, Mexico City, Mexico). Both strains were grown overnight in Luria Bertani (LB) medium (Sigma, St. Louis, MO, USA) at 37°C with shaking, diluted 1:30 in fresh LB medium, and cultured to the logarithmic phase. At an OD of 0.6 at 540 nm, the bacterial concentration was adjusted to obtain the number of bacteria desired. To obtain dead *S. enterica* serovar Typhimurium SL3261, the bacteria were killed by fixation with 4% formaldehyde for 2 h at room temperature. The fixed bacteria were centrifuged at 13,000 rpm for 5 min and washed with PBS to remove the formaldehyde. Killing was confirmed by the lack of bacterial growth on LB plates. On the indicated days post-infection (p.i.), the mice were euthanized, and tissue samples/organs were collected in PBS. The samples were homogenized in PBS, and the cell viability of single-cell suspensions was determined using a TC10TM Automated Cell counter (Bio-Rad, Hercules, CA, USA). An aliquot of the suspension was lysed with 2% Triton X-100 (Sigma Aldrich, San Luis, MO, USA) in 1× PBS, and the number of colony forming units (CFUs) was determined by plating 100 μl of the suspension on LB agar plates (Sigma) and culturing at 37°C overnight.

### Flow cytometry

Single-cell suspensions of thymus tissue were obtained from the infected mice, and specific antibodies were used to immunophenotype the distinct thymocyte subsets. Briefly, RBCs were removed by ammonium chloride treatment. Prior to immunostaining, total thymocytes were pelleted, and γ-globulins were added to block the Fc receptors. The thymocytes were resuspended in PBS with 2% FBS- (Thermo scientific, South Logan, Utah, USA) and stained with anti-CD3 Pacific blue (PB)-, anti-CD44 PE-, anti-CD25 PECy5-, anti-CD69 PECy7-, anti-Vβ4 PE-, anti-Vβ14 FITC-, anti-Vβ3 PE-, anti-Vβ5 Allophycocyanin (APC)- (BD Pharmingen, Franklin Lakes, NJ, USA), anti-CD4 PE-Alexa 610- (Invitrogen, Grand Island, NY, USA), anti-CD8 APC-Cy5.5- (Biolegend, San Diego, CA, USA), and anti-Vβ8.1/8.2 FITC- (eBioscience, San Diego, CA, USA) labeled antibodies or with the appropriate isotype controls (eBioscience). After antibody staining, the cells were fixed with fresh 2% paraformaldehyde. Three- and four-color flow cytometric analysis of the cells was performed with a Cyan ADP flow cytometer (Beckman Coulter, Brea, CA, USA). To identify infiltrating polymorphonuclear (PMN) cells, the thymus was digested with 0.4 μg/ml collagenase at 37°C for 2 h with frequent agitation. The cell suspension was filtered through a 70-μm nylon mesh and washed once in PBS containing 2% FBS. The cells were stained with anti-CD11c APC-, anti-CD11b PE-Cy7-, anti-Gr1 Alexa700- (BD Pharmingen), anti-F4/80 biotin- (Serotec, Raleigh, NC, USA), and anti-Dec205 APC- (eBioscience) labeled antibodies. Cell viability was assessed by the incorporation of propidium iodide and 7-aminoactinomycin D (7-AAD). The data were analyzed using the Summit 5.1 program (Beckman Coulter).

### Histological examination

Thymic tissues were collected in a 4% formol buffer, embedded in paraffin, sectioned at a thickness of 4 μm, and stained with H&E using routine histological techniques.

### Measurement of in vivo BrdU uptake by *Salmonella*-infected thymi

Mice were pulsed i.p. with 1 mg of BrdU (BD Bioscience, San Jose, CA, USA) per day for 2 days. After 24 h, the mice were sacrificed, and the thymocytes were stained with anti-CD4 and anti-CD8 mAbs. The percentage of thymocytes that incorporated BrdU was quantified via flow cytometry using the BrdU Flow kit (BD Biosciences).

### Antibiotic treatment of infected mice

Ciprofloxacin was administered as a single dose of 50 μg i.p. at day 3 p.i. and then continuously in the drinking water at a concentration of 1 mg/ml until 5 or 30 days p.i.. The water was replaced twice per week with water containing fresh antibiotic.

### Statistical analysis

The data were analyzed using an unpaired one-tailed *t*-test using Prism5 (GraphPad Software, La Joya, CA, USA), and *P*-value ≤ 0.05 was considered significant.

## Results

### *Salmonella* infection leads to total thymocyte decline and disrupts the thymic architecture

**Figure 1:**
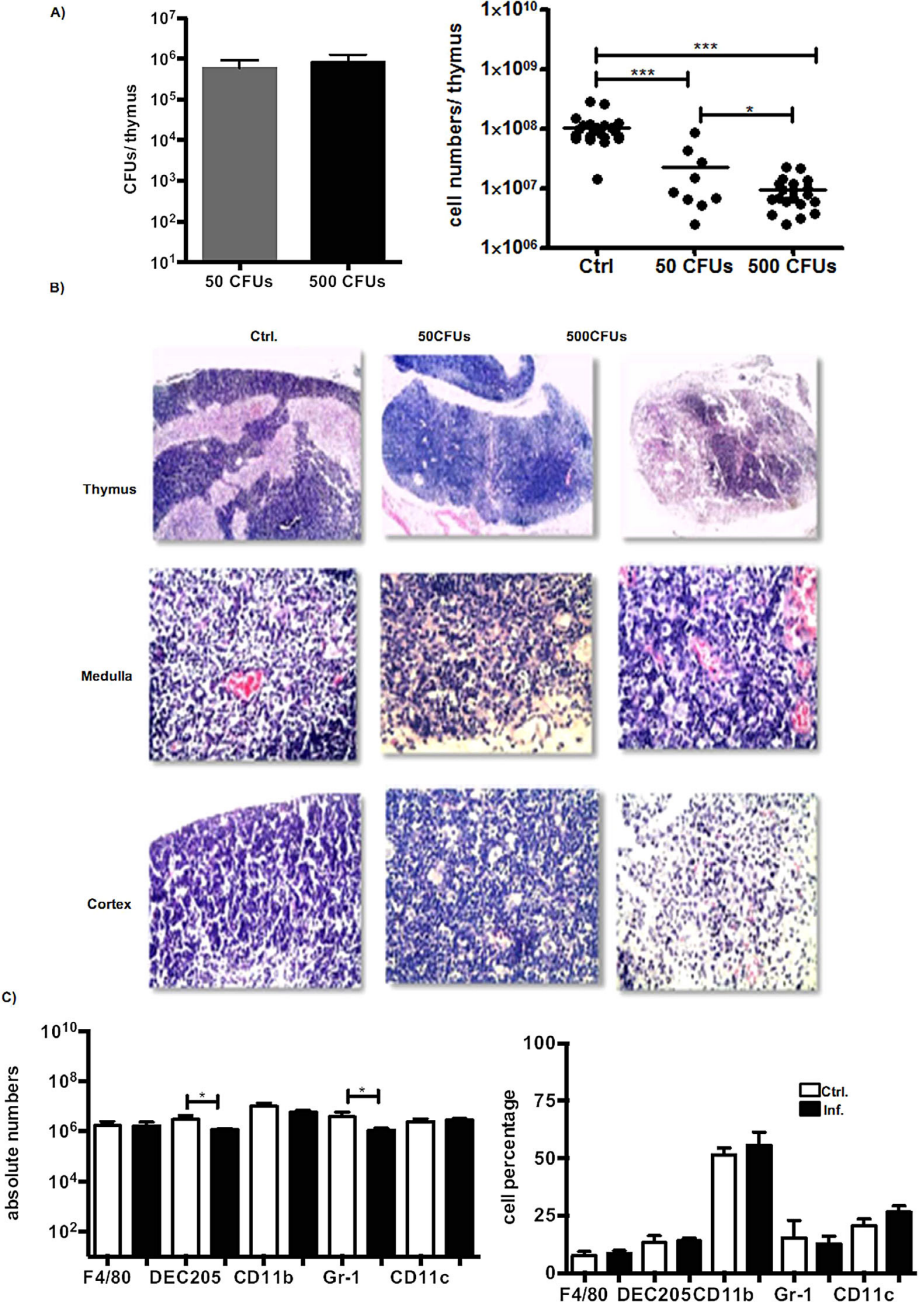
Invasion of *Salmonella* into the thymus leads to a severe reduction in total cell numbers and destruction of the cortical and medullary zones. (A, left) *Salmonella* CFUs from total thymic cells on day 5 after i.p. infection of mice with 50 or 500 MOI of virulent *S. typhimurium* 14028. The data are expressed as the means ± SEM. (A, right) Total thymocyte numbers from mice infected as described previously. The data are average values for five mice from three experiments. *P = 0.02, ***P < 0.0001. (B) H&E staining of thymi from non-infected and infected mice (as described in A). The upper three panels are at 4× magnification, and the lower six are at 40× magnification. (C) Absolute cell numbers and percentages of CD11c-, DEC205-, Gr-1-, CD11b-, and F4/80-positive cells in the thymi of infected mice (as described above). Control (Ctrl) indicates cells from the thymi of non-infected mice. The data are expressed as the means ± SEM. The analyzed PMN cell populations were gated within the more granulocytic region with an increased cell size based on the forward and side scatter parameters of the flow cytometric analysis. The data shown in Figure 1C represent an average of five mice from three independent experiments. *P < 0.05.

Both *Salmonella* infection and mononuclear cell infiltration contribute to inflammation of the reticuloendothelial system, including intestinal Peyer's patches, mesenteric lymph nodes, the liver, and spleen 29,30. To establish whether the damage to the thymi of infected mice is dependent on the bacterial load, we infected mice with 50 (low dose) or 500 (high dose) virulent *S. typhimurium* cells and evaluated changes in the thymic structure and total thymic cell numbers. At day 3 p.i., bacteria were not detected in the thymus and no changes were evident among the thymic populations, regardless of the presence of viable bacteria in the spleens of the infected mice from both groups. However, at day 5 p.i., an increased number of bacteria in the spleen followed by a decrease in the total splenic cell numbers of infected mice was observed with the higher dose of bacteria (Supplementary Fig. S1). Regarding the thymus, a similar number of bacteria were recovered from both groups of infected mice followed by tissue damage and a decline in the total thymic cell numbers (Fig. 1A, B).

In a non-infected thymus, well-defined cortical and medullary structures are evident, with cells that are mainly localized within the cortex. The presence of *Salmonella* in the thymus leads to a reduction in the cellularity of the cortex and cell enrichment of the medullary region. Of note, alterations in the structure and cellularity of the thymus were more pronounced with the higher dose of bacteria (Fig. 1B). Taken together, these results suggest that the number of CFUs in the thymus is independent of the initial infective dose; however, thymic atrophy is dependent on the immune response established by the initial dose of bacteria. To determine the phenotypes of cells present in the thymi of the mice infected with 500 bacterial cells, we treated the infected thymi with collagenase and performed immunostaining for Gr-1, DEC205, CD11b, F4/80, and CD11c at day 5 p.i. As indicated in Figure 1C, the absolute numbers of polymorphonuclear cells were mostly maintained without modifications, except for the DEC205^+^ dendritic and Gr-1^+^ cell populations, which were significantly decreased compared to the non-infected mice. Nonetheless, the frequency of each population was similar between the infected and non-infected mice (Fig. 1C). Thus, the decrease in total thymic cell numbers of the infected mice was not the result of a loss of polymorphonuclear cell populations.

### A *Salmonella*-infected thymus supports the generation of CD4 and CD8 single-positive cells

As mentioned previously, the integrity of the thymic architecture is crucial for proper T-cell development to occur. Therefore, it is important to establish whether the dose-dependent effects observed in the cortical and medullary structures of a *Salmonella*-infected thymus also have a differential impact on T-cell developmental processes.

**Figure 2:**
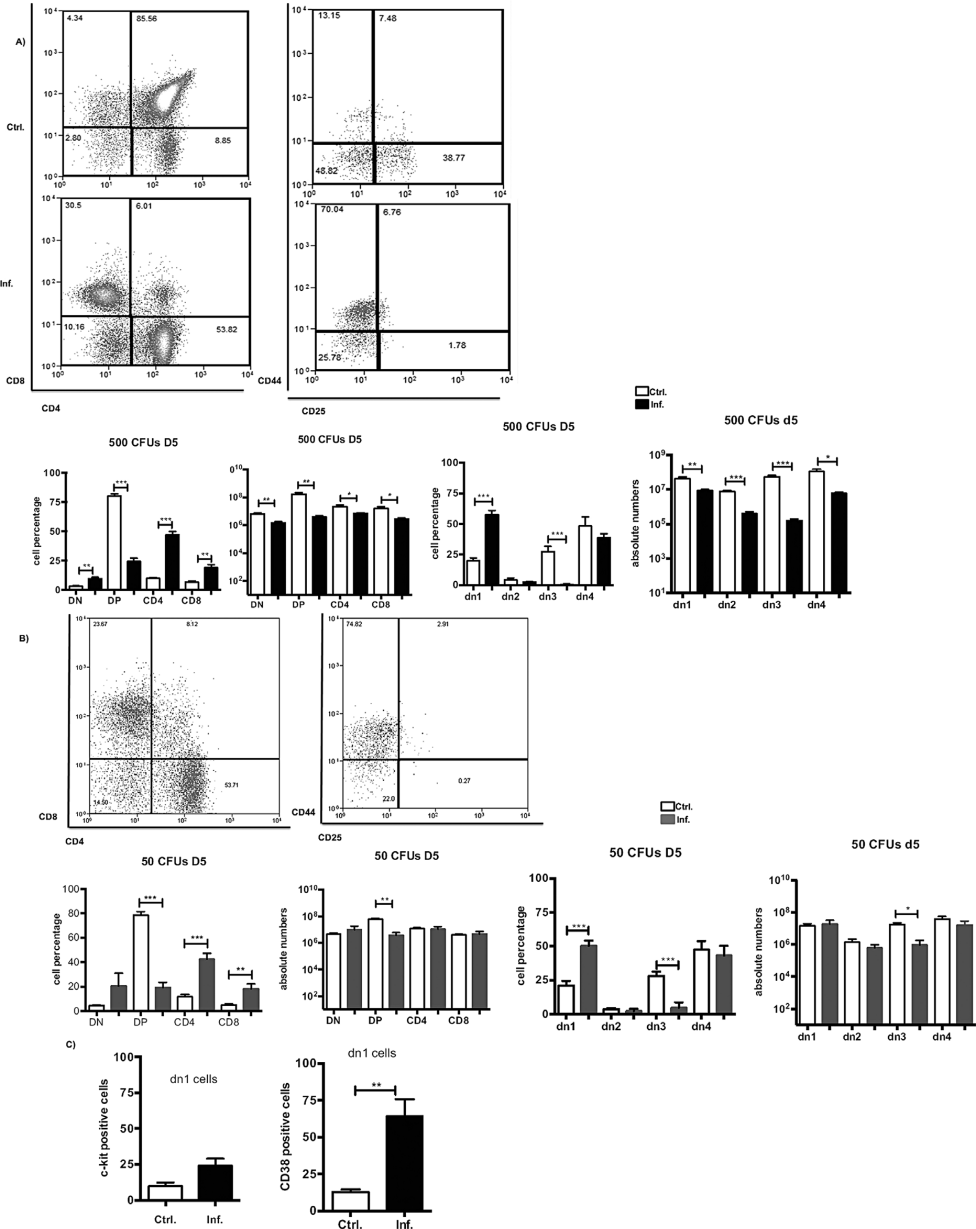
*Salmonella* infection alters thymocyte subpopulations. Thymocyte populations from *Salmonella*-infected mice that received (A) 500 or (B) 50 bacterial cells were quantified via flow cytometry based on the expression of CD4 and CD8 and by CD44 and CD25. The dn1–dn4 cells were gated within the DN region and identified according to CD44 and CD25 expression. (C) Percentage of c-Kit^+^ and CD38^+^ cells gated in the dn1 (CD44^+^CD25^−^) region by flow cytometry. Numbers in the quadrants indicate the percentages of cells. The data were obtained 5 days after an i.p. injection with the virulent strain of *S. typhimurium*. The data are representative of three independent experiments with five mice each. The graphs show the means ± SEM. *P < 0.02, **P < 0.005, ***P < 0.0001.

To achieve this goal, we evaluated the presence of distinct thymocyte populations in mice infected with 50 or 500 bacterial cells at 5 days p.i. Under homeostatic conditions, the DP subset represents the main thymocyte population (80%) followed by the SP subset (15%), and DN cells constitute the remaining 5% of the population 1. Within the DN subset, four subpopulations can be identified based on their expression of CD44 and CD25. When the percentages of these thymocyte populations were analyzed, we observed a redistribution of the thymocytes along distinct maturation stages in mice that were infected either with 50 or 500 bacterial cells (Fig. 2A, B). An increase in the DN subset was observed together with a drastic decrease in DP cells, followed by an increase in the CD4 and CD8 single-positive subset (Fig. 2B). In a more detailed analysis of the DN population, we observed a decline in dn3 cell numbers in the mice that were infected with 50 bacterial cells, whereas an overall reduction in all four thymocyte subpopulations (dn1–dn4) was apparent with the higher dose of bacteria.

As reported elsewhere 27, infection with an attenuated strain of *Salmonella* leads to an increase in the arrival of progenitor T cells to the thymus. Therefore, we sought to determine whether the elevated ratios of DN cells corresponded to an enhanced accumulation of T-cell progenitors by immunostaining for the conventional progenitor marker c-kit and the activation molecule CD38. As shown in Figure 2C, we did not observe a significant increase in the c-kit^+^ cell population, but an accumulation of CD38^+^ cells was detected within the dn1 subset in the mice that were infected with 500 bacterial cells.

To evaluate the impact of *Salmonella* virulence on the T-cell distribution dynamics reported herein, we infected mice with 500 bacterial cells of the attenuated *Salmonella* strain Aroa^−^ or *Salmonella* fixed with paraformaldehyde and evaluated the distribution of their thymocytes at 5 days p.i.

As illustrated in Supplementary Figure S2, the dead *Salmonella* barely affected the ratios of the thymocyte populations, with modest reductions in the DP and SP subsets (Supplementary Fig. S2A). In contrast, following infection with the attenuated strain, the distribution of thymocytes favored the enhancement of DN and CD4 SP thymocytes while reducing the DP subset (Supplementary Fig. S2B). These results confirm that the virulence of the *Salmonella* strain affects the T cell developmental process in the thymus.

It is important to define whether the controlled balance between apoptosis and proliferation that occurs in a normal thymus is altered by infection. Previous studies have reported significant cell death of CD4^+^CD8^+^ thymocytes but not of single-positive thymocytes or peripheral lymphocytes during *S. typhimurium* infection 28. Therefore, we evaluated the apoptotic and proliferative status of all the thymocyte subsets during a *Salmonella* infection.

**Figure 3:**
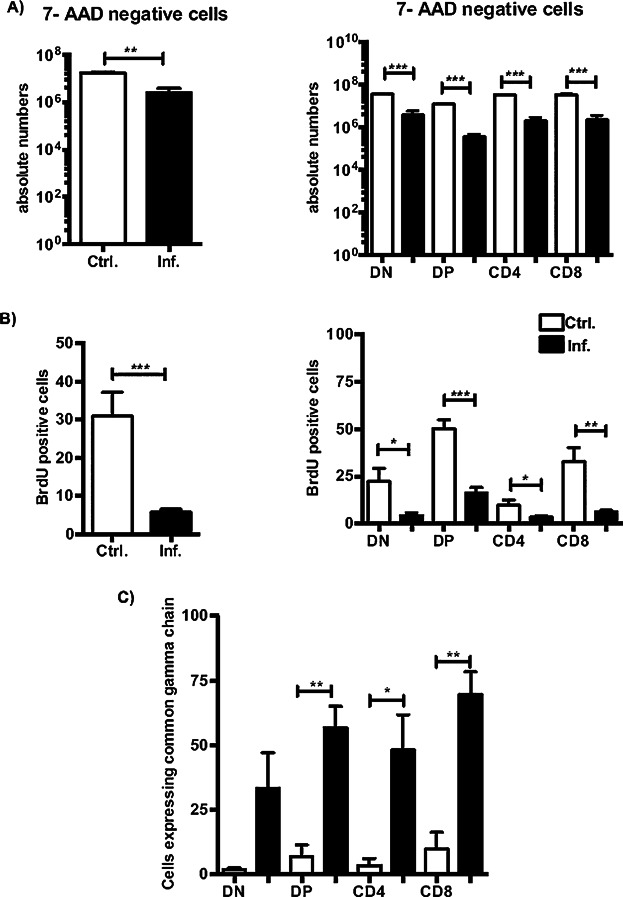
Mature thymocyte populations are maintained during *Salmonella* infection. (A) The absolute numbers of viable cells among the distinct thymocyte populations in the control and infected mice were assessed with 7-AAD staining. (B) The percentage of in vivo BrdU-incorporating thymocytes at day 5 p.i. as assessed by flow cytometry. (C) Expression of the γC receptor among DN, DP and SP thymocyte subsets. The data are expressed as the means ± SEM and represent an average of five mice from three independent experiments. *P < 0.05, **P < 0.005, ***P < 0.0005.

To address cell viability, we stained the thymocyte cell suspension with antibodies directed against CD4 and CD8 followed by co-staining with 7-AAD. As depicted in Figure 3A, there was a significant decrease, but not a complete loss, of viability among all the thymocyte populations. In addition, proliferation was markedly reduced among the distinct thymocyte subsets (Fig. 3B). Notably, when we analyzed the expression of the common gamma chain (γC) receptor, which is an important regulator of proliferation, differentiation, and apoptosis in thymocytes 31–33, we observed a marked increase in the DP and in the CD4 and CD8 SP cells (Fig. 3C). These data suggest that the enhanced proportions of CD4 and CD8 SP cells observed during infection are derived from positive selection.

### Enhanced positive selection of CD4 and CD8 SP thymocytes modifies the T-cell repertoire

**Figure 4:**
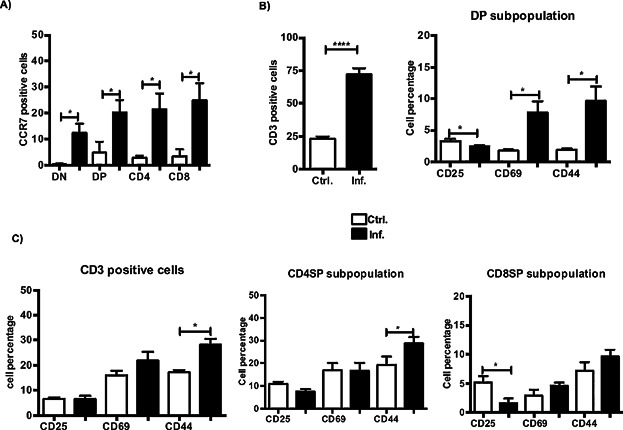
Positive selection of CD4 and CD8 SP thymocytes is increased during infection. (A) Percentage of CCR7^+^ cells within the four distinct thymocyte populations during infection. (B) Quantitative analysis of the total CD3, CD25, CD69, and CD44 expression among CD4^+^CD8^+^ (DP) thymocytes. (C) Expression of CD25, CD44, and CD69 within the mature CD3-positive cells and CD4 and CD8 SP populations gated on CD3. Values in the graphs are expressed as the means ± SEM and represent an average of five mice from three independent experiments. *P < 0.05, ****P < 0.0001.

To determine whether the T-cell maturation process was sustained during the *Salmonella* infection, we analyzed the expression of various markers that were induced in the DP population following the positive selection of these cells. As depicted in Figure 4A, among all the distinct subsets, the overall induction of the chemokine receptor CCR7 supported the cell enrichment observed in the medulla. Furthermore, the heightened total CD3 expression and upregulation of CD69 and CD44 in the DP cells suggested an ongoing T-cell selection process in the infected thymi (Fig. 4B). However, as expected, the newly generated SP T cells were maintained in a non-activated state (Fig. 4C).

**Figure 5:**
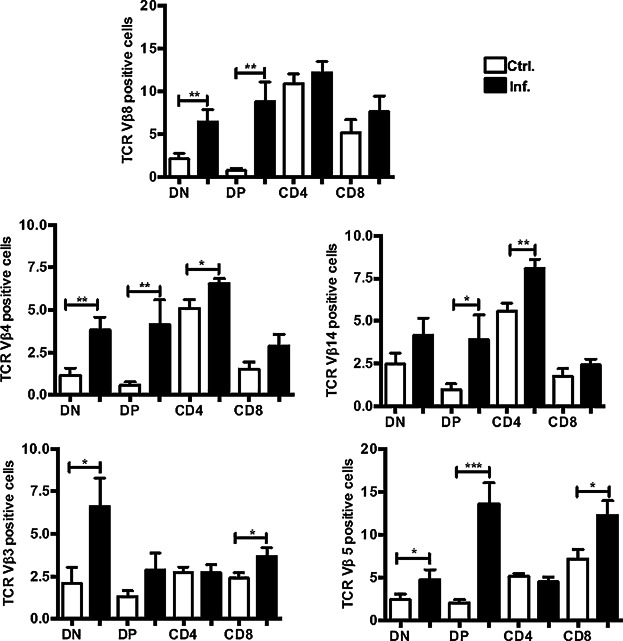
The usage frequency of specific TCR-Vβ chains is modified during infection. The graphs represent the mean percentage ± SEM of the TCR-Vβ chains 8, 4, 14, 3, and 5 in the dn4, DP, and SP populations during infection, as assessed by flow cytometry. The data are from an average of five mice from three independent experiments. *P = 0.02, **P < 0.005, ***P < 0.0001.

To investigate the consequences to T-cell developmental processes of the thymic atrophy induced by *Salmonella*, we examined the TCR repertoire that was generated in the infected mice compared to that in the non-infected mice by analyzing the selection of several TCR-Vβ chains during the transition from DP to SP thymocytes. As presented in Figure 5, the percentage of DP cells expressing the TCR-Vβ8 chain increased during infection. However, this enhancement was not reflected in either the CD4 or CD8 SP compartment; in contrast, the augmented DP TCR-Vβ14^+^ and Vβ4^+^ cells developed toward a CD4 SP phenotype, whereas DP TCR-Vβ3^+^ and Vβ5^+^ cells were committed to a CD8 SP lineage. Regarding other Vβ chains that were not assessed, we believe that some of these chains might also undergo changes similar those observed above.

In conclusion, the positive selection induced by a *Salmonella* infection favors the generation of particular CD4 and CD8 SP T-cell clones, thus modifying the normal TCR usage frequency.

### Antibiotic bacterial control reverses thymic atrophy and re-establishes the T-cell selection process

**Figure 6:**
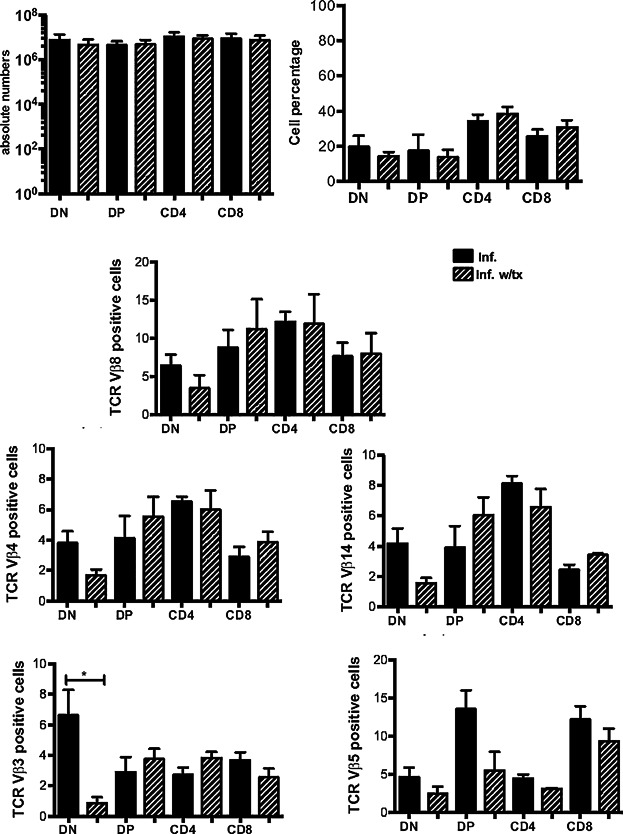
Short-term antibiotic treatment maintains alterations induced by *Salmonella* infection in the thymus. Infected mice were treated with ciprofloxacin as described in the Materials and Methods section and evaluated for (A) modifications in thymocyte distribution and (B) specific TCR-Vβ chain selection in the dn4, DP, and SP populations. The data represent average values for five mice from three independent experiments. *P < 0.05.

**Figure 7:**
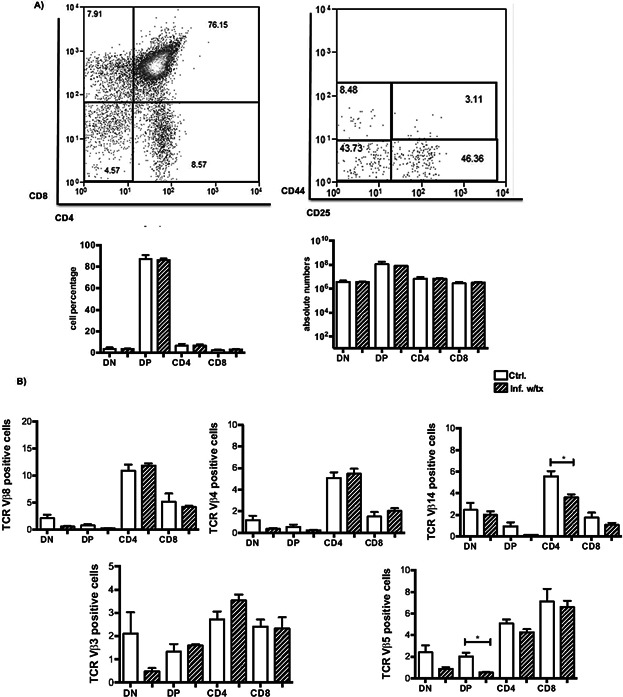
The frequency of specific TCR-Vβ chains that is modified during infection is reversed with long-term antibiotic treatment. (A) Representative flow cytometric dot plot of the four thymocyte subsets (based on the expression of CD4 and CD8) in the infected mice at 30 days p.i. The percentages of thymocytes in the four DN subsets (dn1–dn4) were gated within the DN region based on the CD44 and CD25 expression. The numbers in the quadrants indicate cell percentages within each region. Absolute numbers and the percentages are shown below the dot plots. (B) Flow cytometric analysis indicating the mean percentages ± SEM of cells expressing the TCR-Vβ chains 8, 4, 14, 5, and 3 in the dn4, DP, and SP subsets. *P < 0.05.

**Figure 8:**
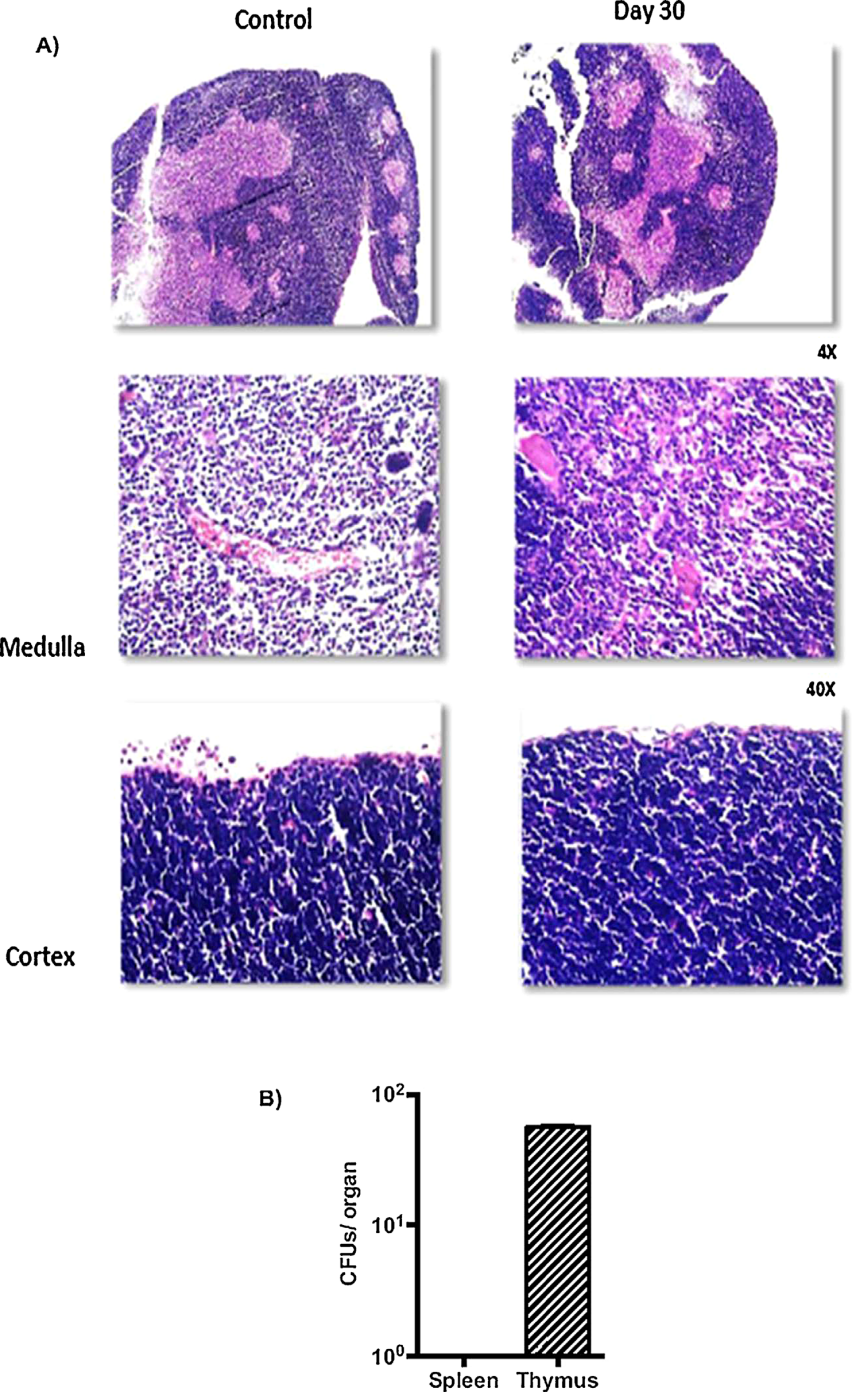
Thymus atrophy in infected mice is reversed with long-term antibiotic treatment. (A) Immunohistochemistry of H&E-stained thymi from non-infected and infected mice after treatment for 30 days with 1 mg/ml ciprofloxacin. (B) The mean ± SEM number of *Salmonella* CFUs recovered from the total spleens or thymi of infected mice after treatment with ciprofloxacin for 30 days. Representative images from three independent mice are shown. Magnification, 4× for the upper two panels and 40× for the lower four panels.

Finally, it is important to establish whether defects observed during *Salmonella* infection are imprinted on the thymus after the infection is resolved. Therefore, the infected mice were treated with 50 μg of the antibiotic ciprofloxacin by i.p. injection at 3 days p.i. followed by a continuous low-dose treatment in the drinking water up to 5 or 30 days p.i. As shown in Figure 6, after 2 days of antibiotic treatment, the infected mice maintained an altered thymocyte distribution together with the skewed TCR repertoire. However, with the long-term treatment of the infected mice, the thymocyte distribution and unaltered TCR repertoire were restored (Fig. 7A, B). Regarding the thymic structure, the cellularity of the cortical and medullary regions was comparable to that in the non-infected mice (Fig. 8A). Nevertheless, despite the elimination of bacteria from the spleen, viable bacteria were recovered from the thymi of the infected mice after 1 month of ciprofloxacin treatment (Fig. 8B). This last observation highlights a novel function of the thymus as a potential reservoir organ for the persistence of *Salmonella*.

## Discussion

In the present study, we demonstrated that *Salmonella* infects the thymus, leading to tissue remodeling and augmentation of the selection of CD4 and CD8 SP cells with particular TCR-Vβ chains. Moreover, these alterations were reversed following the administration of an antibiotic. Previous reports have indicated that both macrophages and DCs are targets for *Salmonella* infection 34,35. In addition, DCs are known to continuously migrate to the thymus for the presentation of captured auto-antigens 36. Therefore, the invasion of *Salmonella* into the thymus may be a consequence of the infiltration of the organ with PMN cells. We demonstrated that *Salmonella* infection in the thymus had a more drastic effect on the total thymic cell numbers than on the PMN cell population (Fig. 1). In addition, the structure of the thymus was more severely affected by a higher dose of bacteria regardless of the number of bacterial cells that were present in the organ. This observation suggests that tissue damage and thymocyte subset alterations depend on the initial innate immune cell priming events that occur in the periphery during infection.

Moreover, the alteration of the thymic structure as well as the interaction between potentially infected DCs and DP thymocytes may contribute to the defects in the thymocyte distribution and apoptotic and proliferative processes observed during infection. Therefore, we assessed the effects of *Salmonella* invasion on the generation of CD4 and CD8 SP cells. As mentioned previously, a profound depletion of DP thymocytes has been reported during *Salmonella* infection and has been attributed to increased levels of cortisol in the sera of the infected animals. Our results confirmed an enhanced apoptosis, but not complete absence, of all the distinct thymocyte populations. Taken together, our findings support the hypothesis that the pathways utilized by endogenous glucocorticoids and inflammatory cytokines, such as IFN-γ, synergize to enhance the death of immature thymocytes during *S. typhimurium* infection 28. Furthermore, the proliferation of all the thymocytes was diminished during infection, although the expression of the γC receptor was enhanced, suggesting that the activation of survival signaling pathways contributed to the maintenance of the thymocyte populations. However, it is also important to consider that the lack of an interaction between the thymocytes and cortical epithelial cells due to thymic atrophy may impact the proliferation and survival capacity of the thymocytes 11. Nevertheless, during the positive selection of thymocytes, the apoptotic signaling that is induced during negative selection is abrogated. We observed a sustained T-cell developmental process, as measured by the increase in CD69 and CD44 molecules, which, in turn, sustained the selection of CD4 and CD8 SP thymocytes from particular TCR-Vβ families. As expected, the increase in CD69 was restricted to the DP population and was reduced in the selected CD4 and CD8 SP cells. This finding is in agreement with previous reports that have established a controlled inhibition of the activation state of thymocytes during infection 26,37. In addition, the overall upregulation of CCR7 indicated that the thymocytes, including the DN cells, were recruited to the medullary zone, which was found to be the least affected area.

It remains to be established whether the selected T-cell clones generated during *Salmonella* infection can recognize tissue-specific antigens or even other *Salmonella* antigens. However, it is important to consider that the SP thymocytes that are selected during infection are normally submitted for apoptosis under homeostatic conditions; therefore, the generation of these cells could potentially represent a harmful autoimmune response. Consequently, it was important to establish whether the skewed T-cell selection process was conserved following control of the *Salmonella* infection. In the present study, infected mice were thus treated with the antibiotic ciprofloxacin up to 5 (short term) or 30 (long term) days p.i., and then the thymic architecture and thymocyte subset distribution were assessed. Short-term antibiotic treatment did not alter the thymocyte distribution or the TCR-Vβ usage frequency observed during infection. However, long-term (30 day) antibiotic treatment resulted in the re-establishment of the thymocyte distribution, and the selection of CD4 and CD8 SP cells was comparable to that observed in the uninfected controls. Regarding the architecture of the thymus, the alterations of the cortical and medullary structures were reversed by the antibiotic treatment. It remains to be established whether the use of antibiotic treatment induces T-cell clones to undergo pro-apoptotic signaling or, more importantly, to be released into the periphery. Experimental evidence from studies investigating *Trypanosoma cruzi* infection has demonstrated that T cells expressing “forbidden” TCRs, in particular the Vβ5 and Vβ12 families, survive and can be detected in peripheral lymph nodes 38. Nonetheless, because of the non-enrichment of thymic SP cells, the presence of these forbidden TCRs in the periphery is attributed to an abnormal migration of immature cells. Finally, although bacteria were eliminated from the spleen, we were able to recover *Salmonella* from the thymi of infected mice after long-term antibiotic treatment. This finding highlights the importance of the thymus as a potential reservoir organ that permits the persistence of *Salmonella*.

## Supporting Information

Figure S1 . Dynamics of *Salmonella* infection in the spleen with different bacterial doses. C57BL/6 mice were infected with 50 or 500 bacterial cells of S. *typhimurium* virulent strain 14028 and assessed at 3 and 5 days p.i. for CFUs in the spleen (upper graphs) and total splenic cell numbers (lower graphs). Graphs show the means ± SEM for five mice from three independent experiments. **P* < 0.05.

Figure S2 . *Salmonella* pathogenesis differentially affects the subset distribution of thymocyte cells. (A) C57BL/6 mice were infected with 500 bacterial cells of virulent *Salmonella* fixed with paraformaldehyde or (B) the attenuated *Salmonella* Aroa^−^ strain and evaluated 5 days p.i. for changes in absolute numbers and the percentages of distinct thymocyte subsets (based on the expression of CD4 and CD8). The graphs show the means ± SEM for five mice from three independent experiments. **P* < 0.05, ***P* < 0.005, ****P* ≤ 0.0005.
