# Supplementary figures and images for: Bacterial clearance reverses a skewed T-cell repertoire induced by Salmonella infection
Source: Immun Inflamm Dis. 2015 May 6;3(3):209–23. doi: 10.1002/iid3.60 (PMC4578521; doi:10.1002/iid3.60)

Supplementary Figure 1

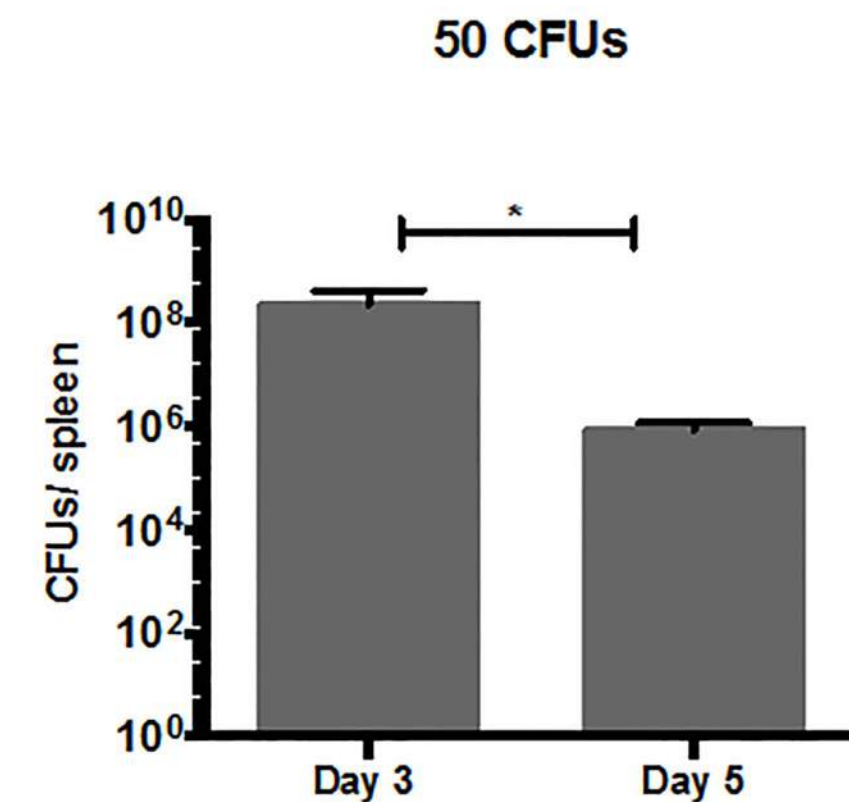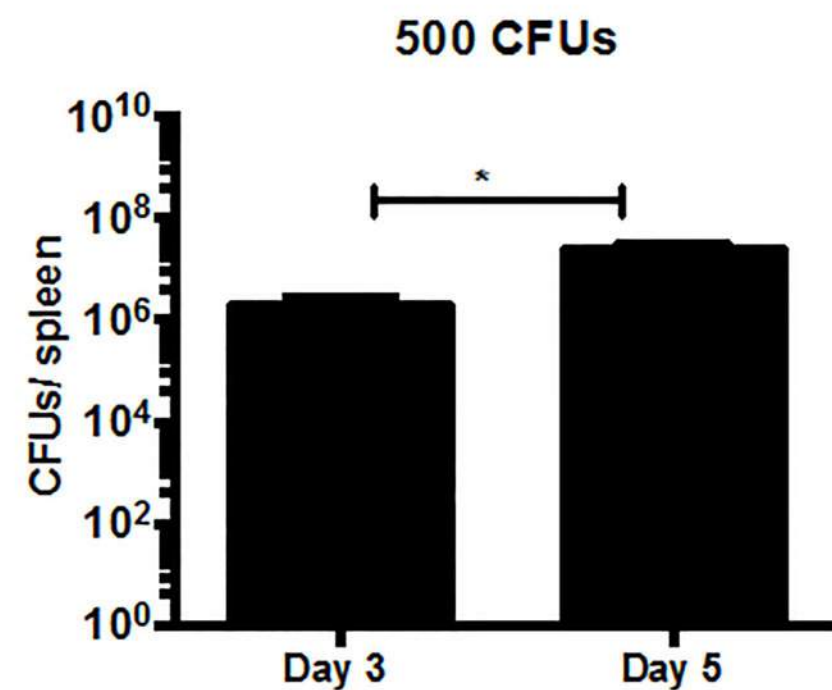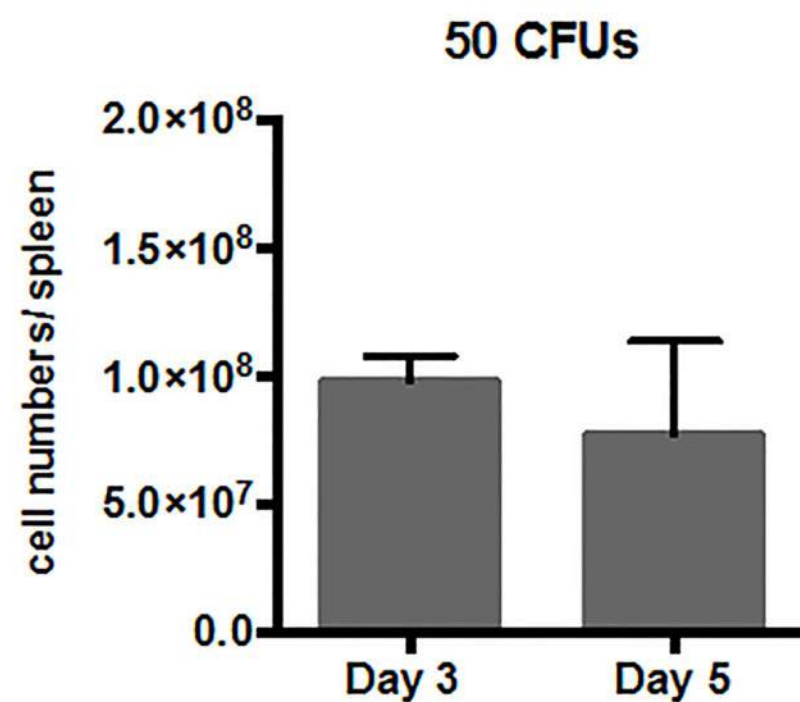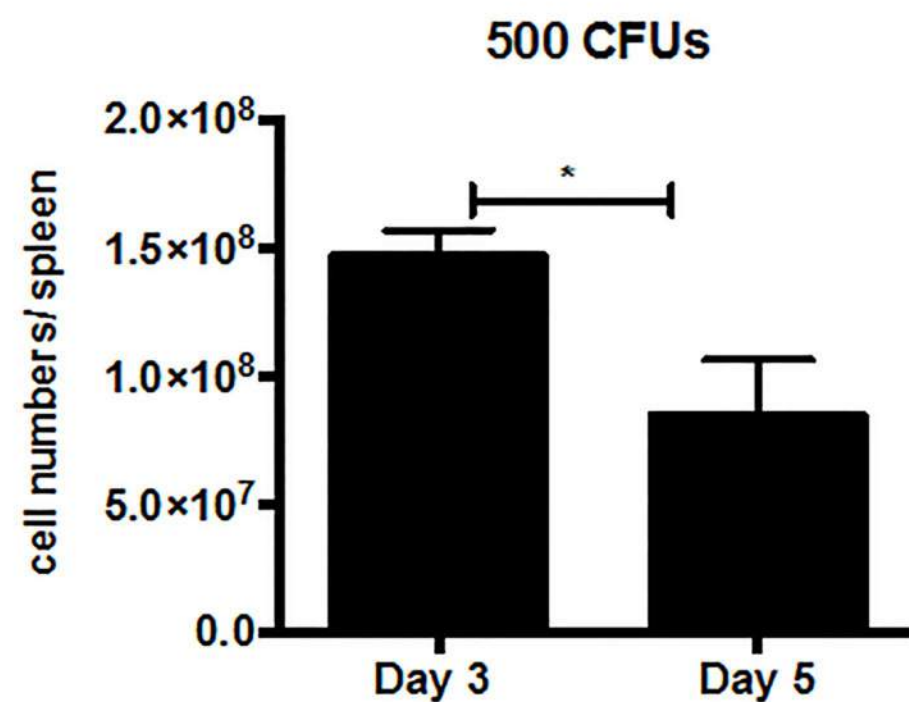

Supplement: Figure S1 — . Dynamics of Salmonella infection in the spleen with different bacterial doses. C57BL/6 mice were infected with 50 or 500 bacterial cells of S. typhimurium virulent strain 14028 and assessed at 3 and 5 days p.i. for CFUs in the spleen (upper graphs) and total splenic cell numbers (lower graphs). Graphs show the means ± SEM for five mice from three independent experiments. *P < 0.05. [file iid30003-0209-sd1.pdf]

Supplementary Figure 2

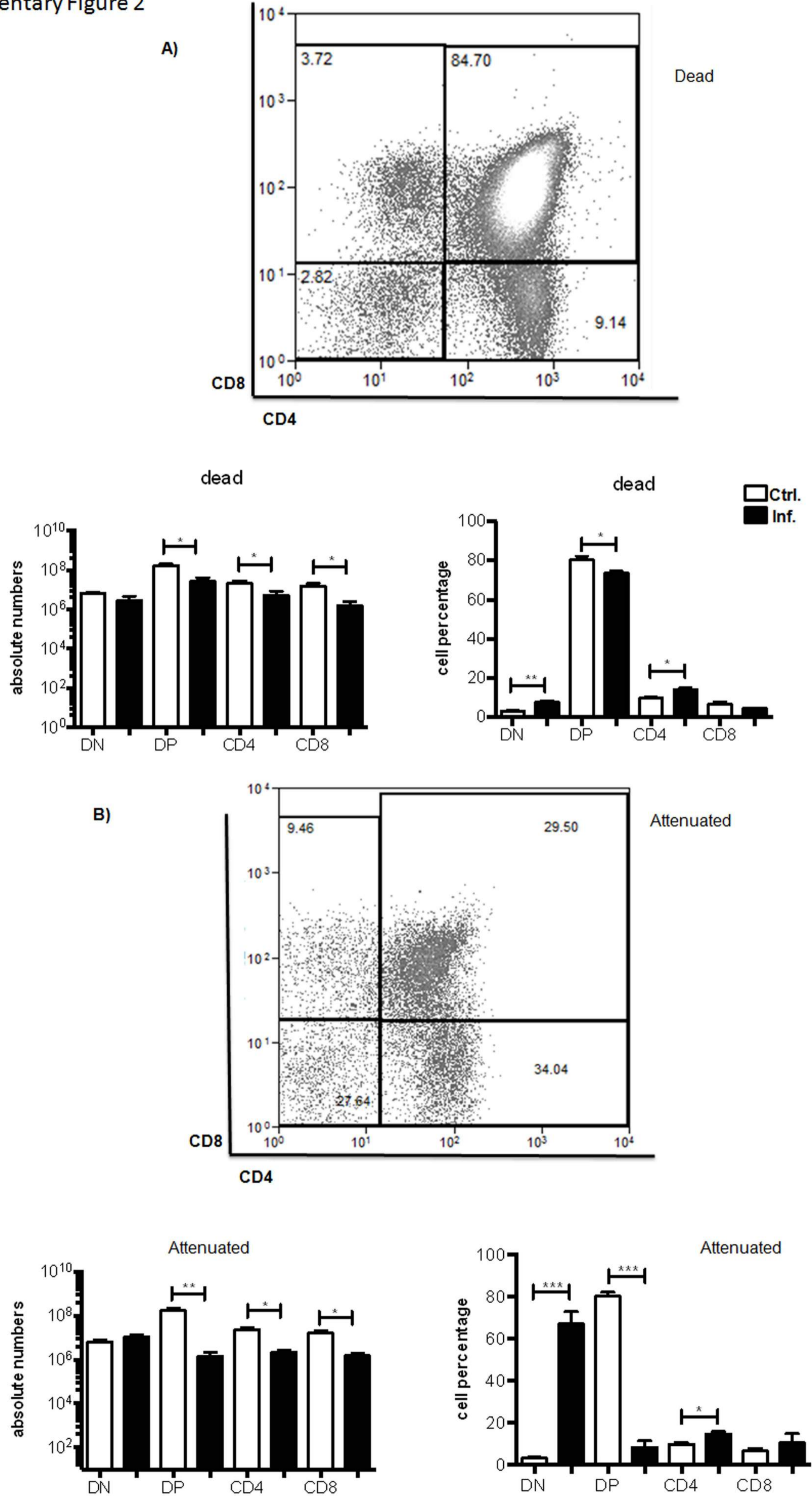

Supplement: Figure S2 — . Salmonella pathogenesis differentially affects the subset distribution of thymocyte cells. (A) C57BL/6 mice were infected with 500 bacterial cells of virulent Salmonella fixed with paraformaldehyde or (B) the attenuated Salmonella Aroa− strain and evaluated 5 days p.i. for changes in absolute numbers and the percentages of distinct thymocyte subsets (based on the expression of CD4 and CD8). The graphs show the means ± SEM for five mice from three independent experiments. *P < 0.05, **P < 0.005, ***P ≤ 0.0005. [file iid30003-0209-sd2.pdf]
